# Composition and Acidification of the Culture Medium Influences Chronological Aging Similarly in Vineyard and Laboratory Yeast

**DOI:** 10.1371/journal.pone.0024530

**Published:** 2011-09-19

**Authors:** Christopher J. Murakami, Valerie Wall, Nathan Basisty, Matt Kaeberlein

**Affiliations:** Department of Pathology, University of Washington, Seattle, Washington, United States of America; Duke University, United States of America

## Abstract

Chronological aging has been studied extensively in laboratory yeast by culturing cells into stationary phase in synthetic complete medium with 2% glucose as the carbon source. During this process, acidification of the culture medium occurs due to secretion of organic acids, including acetic acid, which limits survival of yeast cells. Dietary restriction or buffering the medium to pH 6 prevents acidification and increases chronological life span. Here we set out to determine whether these effects are specific to laboratory-derived yeast by testing the chronological aging properties of the vineyard yeast strain RM11. Similar to the laboratory strain BY4743 and its haploid derivatives, RM11 and its haploid derivatives displayed increased chronological life span from dietary restriction, buffering the pH of the culture medium, or aging in rich medium. RM11 and BY4743 also displayed generally similar aging and growth characteristics when cultured in a variety of different carbon sources. These data support the idea that mechanisms of chronological aging are similar in both the laboratory and vineyard strains.

## Introduction

A model of post-mitotic cellular aging, referred to as chronological aging, has been developed in the budding yeast *Saccharomyces cerevisia*e [1], [2]. In this system, cells are induced to enter a non-dividing state and are maintained in this state for several weeks. The viability of the population over time is measured by quantifying the fraction of the cells that retain the ability to re-enter the cell cycle in the presence of appropriate growth cues.

The most commonly used protocol for measuring chronological life span (CLS) involves culturing cells under aeration in liquid synthetic defined medium with an initial glucose concentration of 2% [1], [2]. The cells undergo a fermentative exponential growth phase in which ethanol is produced from glucose, followed by a metabolic shift to respiration upon glucose depletion. During the shift to respiratory growth, expression of many different mitochondrial enzymes is enhanced to facilitate utilization of ethanol as a carbon source. Within a few days, most of the ethanol has been depleted and the yeast cells exit the cell cycle. Viability over time can then be monitored by removing a small aliquot of cells and plating for colony forming units (CFUs) on rich growth medium (YEPD). A higher-throughput method for quantifying viability during chronological aging has also been developed in which the relative proportion of viable cells is determined based on outgrowth kinetics in YEPD [3], [4].

Several studies have indicated that chronological aging is accompanied by an increasing burden of oxidative stress and an induction of the yeast apoptotic-like response [5], [6]. Damaged proteins and macromolecules accumulate with chronological age, and stress response pathways have been shown to play a central role in chronological longevity [7], [8], [9], [10]. At least some of the genetic factors that modulate chronological life span play a similar role in multicellular eukaryotes, suggesting that aspects of this yeast aging model are conserved [11].

Recently, we reported that accumulation of acetic acid in the culture medium limits the chronological life span of yeast cells aged in synthetic defined medium [12]. Buffering the medium to pH 6–7 significantly extends chronological life span under these conditions [12], [13] and reduces oxidative stress [14]. Growth in rich YEPD medium also results in reduced acidification and extended chronological life span in laboratory strains, relative to strains grown in synthetic defined medium [15]. Life span extension from dietary restriction by reducing the initial glucose concentration can be explained, at least in part, by a corresponding decrease in acetic acid production and medium acidification [12].

Studies of chronological aging, including those described above on acetic acid toxicity, have generally been performed in laboratory yeast strains. The genetic history of such strains is often poorly characterized and most have been subjected to evolutionary pressures to adapt to laboratory conditions that are substantially different from the natural environment of a yeast cell. Evolution in the laboratory could conceivably influence the aging properties of these strains. We therefore set out to examine chronological aging in a yeast strain more recently derived from the natural environment and which has undergone fewer passages in a laboratory setting.

Here we report an initial characterization of chronological aging in the vineyard isolate, RM11, and compare it to a common laboratory strain BY4743 [16]. RM11 is a diploid derivative of a natural isolate collected from a California vineyard [17]. Similar to BY4743, dietary restriction significantly increases the chronological life span of RM11 cells in synthetic defined medium, as does buffering the pH of the medium. Growth and aging in rich medium attenuates the culture acidification and extends chronological life span of both strains, relative to synthetic defined medium. Similar results were obtained with haploids of both mating types derived from the vineyard and laboratory strain. These data in combination with prior studies suggest that growth under laboratory conditions has not significantly altered the way that yeast chronologically age, at least under the standard conditions currently used by most laboratories. They also suggest that acidification of the medium and acetic acid toxicity limit chronological life span to a similar extent in the vineyard and laboratory strains.

## Results

**Figure 1:**
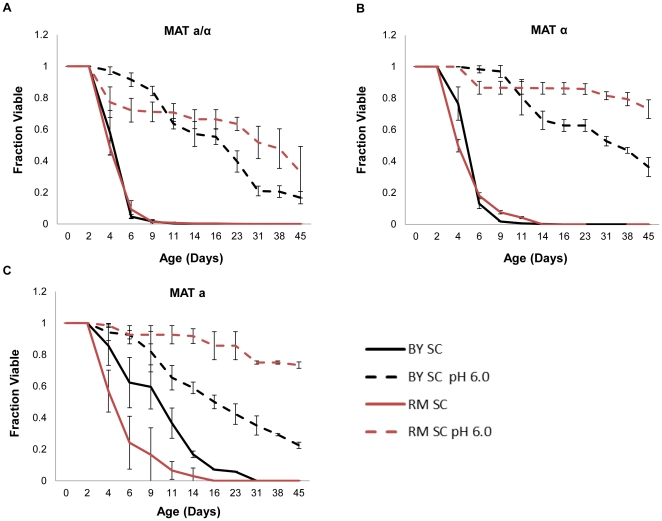
Adjusting synthetic complete medium to a pH of 6.0 extends chronological life span. Life span extension from buffering at pH 6.0 using a citrate phosphate buffer was statistically significant (p<0.05) in (**A**) diploid, (**B**) haploid mating type *α*, and (**C**) haploid mating type **a** of BY4743 (BY) and RM11 (RM). Error bars indicate standard deviation across biological replicates. Corresponding survival integral and p-values are shown in Table 2.

The diploid laboratory strain BY4743 acidifies the growth medium during chronological aging in synthetic defined medium; preventing acidification by buffering the pH of the medium to 6.0 significantly enhances chronological life span [12]. Like BY4743, diploid cells of the vineyard isolate RM11 also acidified the growth medium during chronological aging (**Table 1**). After 48 hours of culture, the pH for both diploid strains had dropped from an initial value of 4.0 to approximately 2.8. Also similar to BY4743, buffering the synthetic complete medium with a pH 6.0 citrate-phosphate buffer significantly extended the chronological life span of diploid RM11 cells (**Figure 1A**). Buffering increased the survival integral (SI), defined here as the area under the survival curve between day 2 and day 45, from 2.5 to 23.1 (p = 2.0×10^−5^) for diploid RM11 and from 2.3 to 19.4 for BY4743 (p = 5.8×10^−5^). Haploid isolates of both diploid strains behaved similarly (**Figure 1B, C**).

**Table 1:** Culture pH of laboratory and vineyard yeast in synthetic defined medium.

Strain	SC 2% Glu	SC 0.5% Glu	SC 0.05% Glu	SC 2% Glu+Cit-Phos Buffer	YEPD
BY4743	2.62	3.26	6.06	4.99	4.18
BY4742	3.01	3.68	6.24	5.16	4.42
BY4741	3.41	4.66	6.18	5.66	5.04
RM11 diploid	2.65	3.18	5.36	5.14	4.52
RM11 *MAT* **a**	2.68	3.34	5.26	5.11	4.47
RM11 *MATα*	2.67	3.34	5.41	5.13	4.51

Cells were grown in synthetic complete medium (SC) with the indicated carbon source or rich YEPD medium, and pH was determined after 48 hours. For growth in buffered medium (Cit-Phos buffer), a citrate phosphate buffer (64.2 mM Na2HPO4 and 17.9 mM citric acid) adjusted to pH 6.0 was added to the medium prior to inoculation. Initial pH values for synthetic complete and YEPD were 4.0 and 6.6, respectively.

**Figure 2:**
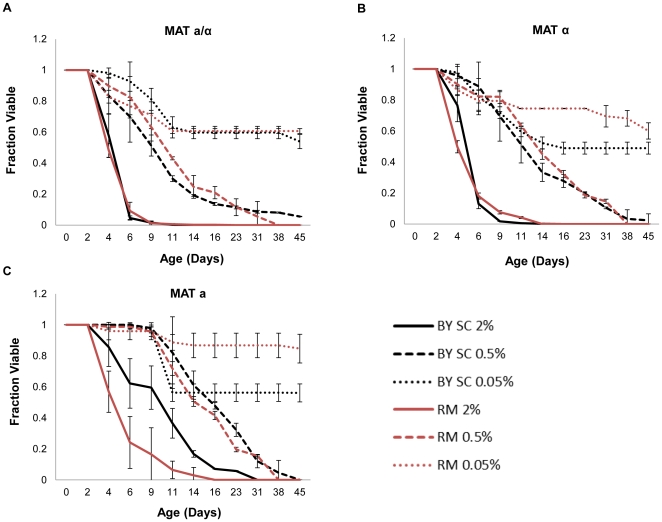
Dietary restriction extends life span in both BY4743 (BY) and RM11 (RM) strain backgrounds. Glucose was reduced to either 0.5% or 0.05% in synthetic medium in (**A**) diploid, (**B**) haploid mating type *α*, and (**C**) haploid mating type **a**. Error bars indicate standard deviation across biological replicates. Corresponding survival integral and p-values are shown in Table 2.

Dietary restriction by reducing the glucose concentration of the culture medium from 2% to 0.05% causes the pH of the growth medium to increase during chronological aging and increases chronological life span in BY4743 [12]. This level of dietary restriction had a similar effect on medium acidification (**Table 1**) and chronological life span in RM11 (**Figure 2A** **;** **Table 2**). Both diploid RM11 and BY4743 cells grown in 0.05% glucose synthetic complete medium had culture pH of approximately 5.7 after 48 hours. Haploid isolates of both diploid strains behaved similarly (**Figure 2B, C** **;** **Table 2**). An intermediate level of dietary restriction (0.5% glucose) also reduced acidification of the culture medium (**Table 1**) and significantly increased chronological life span in all strains examined (**Figure 2** **;** **Table 2**).

**Table 2:** Survival integral (SI) values for BY and RM haploid and diploid strains aged in different medium compositions with glucose as the carbon source.

Medium	Strain	SI (SD)	p-value	Strain	SI (SD)	p-value
SC 2%	BY4741 (MATa)	8 (0.8)		RM11 (MATa)	3.4 (1.3)	
SC 0.5%		17.4 (1.7)	1.0E-03		15.4 (0.4)	1.1E-04
SC 0.05%		27.6 (2)	9.6E-05		38.0 (3.6)	8.2E-05
SC pH 6.0		21.2 (1)	5.8E-05		35.9 (1.5)	9.2E-06
YEP 2%		28.6 (1)	9.9E-06		24.9 (12.5)	4.0E-02
YEP 0.5%		7.8 (0.3)	4.4E-06		17.5 (0.6)	0.4
YEP 0.05%		15.6 (1.9)	4.7E-04		19.7 (2.9)	0.5
SC 2%	BY4742 (MATα)	2.9 (0.3)		RM11 (MATα)	2.7 (0.1)	
SC 0.5%		12.8 (1)	8.7E-05		13.6 (0.7)	1.1E-05
SC 0.05%		24.3 (1.4)	1.1E-05		35.3 (6.7)	1.1E-03
SC pH 6.0		27.6 (1.2)	3.9E-06		36.2 (1.3)	1.6E-06
YEP 2%		26.3 (2.3)	6.6E-05		27.8 (1.7)	9.1E-03
YEP 0.5%		8.9 (1.1)	3.1E-04		16.8 (0.3)	3.4E-04
YEP 0.05%		15.9 (1.6)	3.0E-03		23.1 (1)	1.4E-02
SC 2%	BY4743 (diploid)	2.3 (0.3)		RM11 (diploid)	2.5 (0.2)	
SC 0.5%		9.8 (0.8)	1.4E-04		9.3 (0.8)	1.6E-04
SC 0.05%		28.1 (1.9)	2.0E-05		33.5 (3.8)	1.4E-04
SC pH 6.0		19.4 (1.5)	4.7E-05		23.1 (1.5)	2.0E-05
YEP 2%		17.9 (2.6)	5.1E-04		22 (7.1)	8.6E-04
YEP 0.5%		7.8 (0.4)	2.7E-03		9.5 (1)	4.0E-02
YEP 0.05%		12.4 (0.1)	2.2E-02		19.3 (2.2)	0.6

The % refers to the amount of glucose present in the culture medium. Survival integral values represent the average of three biological replicates between days 2 to 45 in the experiment. Values in parentheses are standard deviation. P-value is calculated by Student's T-test for each conditions relative to synthetic complete (SC) 2% for that genotype, except for YEP 0.5% and YEP 0.05%, which are calculated relative to YEP 2%.

**Figure 3:**
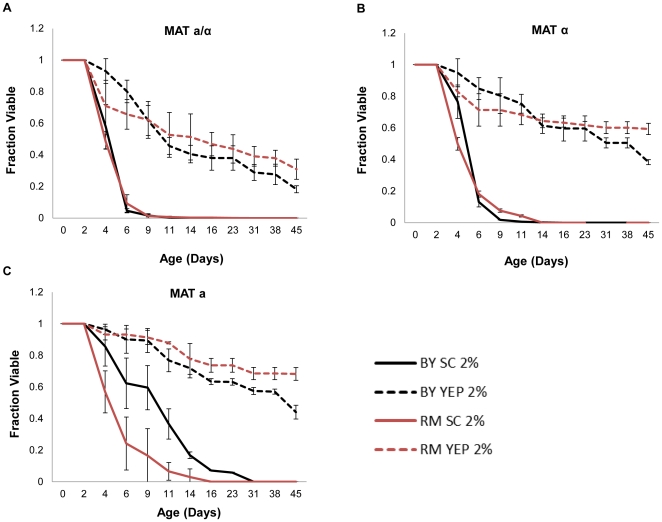
Cells aged in YEPD medium are longer lived than cells aged in synthetic complete medium. Longer chronological life span was observed in rich YEPD medium relative to synthetic complete (SC) in (**A**) diploid, (**B**) haploid mating type *α*, and (**C**) haploid mating type **a** for both BY4743 (BY) and RM11 (RM) strain backgrounds. Error bars indicate standard deviation across biological replicates. Corresponding survival integrals and p-values are shown in Table 2.

It has been previously reported that cells aged in rich YEPD medium live longer than cells aged in synthetic defined medium [1], [2]. Consistent with these data, we find that BY4743 and RM11, as well as all of the corresponding haploid strains, have a longer chronological life span in YEPD medium (**Figure 3** **;** **Table 2**). This longer life span is associated with higher pH of the culture medium after 48 hours (**Table 1**). Dietary restriction by reducing the glucose concentration failed to increase chronological life span in any of the strains aged in rich medium, and, in some cases, reduced life span (**Table 2**).

**Figure 4:**
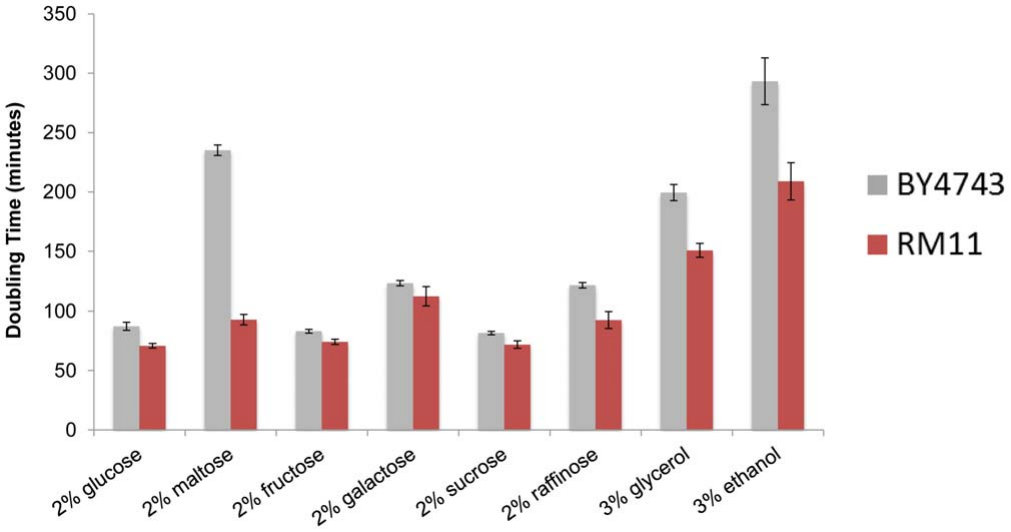
Growth rates of BY4743 and RM11 diploids in the presence of different carbon sources. BY4743 and RM11 were grown overnight in YEPD, diluted 300-fold, and inoculated into YEP containing the indicated carbon source. Error bars represent the standard deviation of five biological replicates.

**Figure 5:**
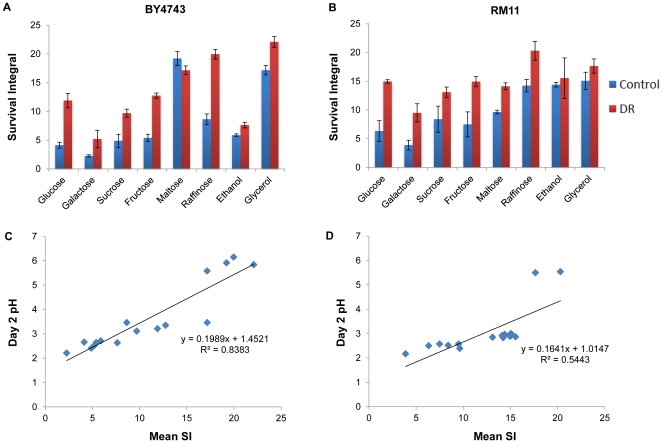
Effect of carbon source on chronological life span correlates with pH of the culture medium after 48 hours of culture. The effect of different carbon sources on chronological life span is shown for diploid (**A**) BY4743 or (**B**) RM11 cells. Cells were aged in synthetic complete medium with the indicated carbon source. Control concentrations for all carbon sources were tested at 2% w/v except ethanol and glycerol which were tested at 3%. Dietary restricted (DR) concentrations were at 0.5% for all sources except ethanol and glycerol which were reduced to 1%. Survival integral (SI) values represent the area under the survival curve between day 2 and day 26 and error bars represent the standard deviation across biological replicates. The survival integral values for growth in different carbon sources correlates significantly with culture pH after 48 hours in diploid (**C**) BY4743 or (**D**) RM11 cells. A similar correlation was observed for pH after 4 days of culture (see Table 4).

In order to further explore the relationship between culture acidification and chronological aging, we carried out chronological life span assays for BY4743 and RM11 diploid cells aged for 26 days in synthetic defined medium with different carbon sources. The following carbon sources were tested: glucose, galactose, sucrose, fructose, maltose, raffinose, ethanol, and glycerol, and the relative ability of each strain to utilize each carbon source was determined by measuring doubling time in rich medium (YEP) supplemented with that carbon source (**Figure 4**, **Table 3**). In general, RM11 showed a slightly faster doubling time and slightly longer chronological life span than BY4742 across the different carbon sources, and the effect of alternative carbon sources on life span was similar between the two strains in most cases: relative to glucose, galactose shortened chronological life span; sucrose and fructose had little effect on life span; maltose, raffinose, and glycerol all significantly extended life span (**Figure 5** **,** **Table 3**). In most cases, dietary restriction, by reducing the amount of carbon source present in the medium, significantly extended life span in both strains. Two notable differences between the strains were the effects of maltose and ethanol on chronological life span. Even at control levels, BY4743 cells aged in maltose-containing media were much longer-lived than cells aged in glucose (**Table 3**). This effect might be explained by the poor utilization of maltose by BY4743 relative to RM11 cells (**Figure 4**). In both strains, however, a significant correlation was observed between chronological life span and culture pH after two days of growth across all carbon sources (**Figure 5**). A similar correlation was observed at day 4 of the experiment (**Table 4**).

**Table 3:** Effect of different carbon sources on culture pH, growth rate, and chronological life span.

BY4743				
Carbon source	Doubling Time (min)	Day 2 pH (control/DR)	Day 4 pH (control/DR)	SI (control/DR)
Glucose	87.31	2.66/3.21	2.54/3.28	4.14/11.90*
Galactose	123.55	2.21/2.52	2.26/2.66	2.27/5.21*
Sucrose	81.58	2.41/3.11	2.73/3.22	4.89/9.70*
Fructose	83.14	2.64/3.35	2.53/3.34	5.40/12.75*
Maltose	235.11	5.91/5.58	6.13/5.87	19.22/17.16
Raffinose	121.85	3.46/6.15	3.43/6.16	8.64/19.97*
Ethanol	292.22	2.71/2.63	2.53/2.60	5.88/7.63*
Glycerol	199.49	3.46/5.84	3.09/5.80	17.17/22.10*

RM11 diploid				
Carbon source	Doubling Time (min)	Day 2 pH (control/DR)	Day 4 pH (control/DR)	SI (control/DR)
Glucose	70.81	2.50/2.88	2.79/2.93	6.33/14.97*
Galactose	112.62	2.17/2.57	2.272.60	3.89/9.50*
Sucrose	71.86	2.52/2.85	2.56/2.94	8.40/13.10*
Fructose	74.23	2.57/2.90	2.36/2.94	7.49/14.93*
Maltose	92.80	2.39/2.92	2.36/2.95	9.64/14.12*
Raffinose	92.59	2.83/5.54	2.75/5.60	14.24/20.31*
Ethanol	208.96	2.97/2.87	2.74/3.84	14.40/15.54
Glycerol	150.84	3.00/5.50	2.63/3.74	15.07/17.65

Cells were aged in synthetic complete medium supplemented with the appropriate carbon source and pH measurements were taken at both day 2 and day 4 of the experiment. Control concentrations for all carbon sources were tested at 2% w/v, except ethanol and glycerol which were tested at 3%. Moderate dietary restriction (DR) concentrations were at 0.5% for all sources except ethanol and glycerol, which were reduced to 1%. Survival integral (SI) values represent the area under the survival curve between day 2 and day 26.

*Denotes a statistically significant increase SI in response to DR (p<0.05).

**Table 4:** Correlation between culture pH and chronological life span.

Strain (day)	Pearson coefficient (r)	p-value (T-test)
BY4743 (day 2)	0.92	<10^−5^
BY4743 (day 4)	0.89	<10^−5^
RM11 (day 2)	0.74	0.001
RM11 (day 4)	0.73	0.001

BY4743 and RM11 cells were aged in synthetic complete medium with different carbon sources as shown in **Table 3** and **Figure 5**. Culture pH was measured at either day 2 or day 4 of the experiment.

## Discussion

The primary goal of this study was to determine whether a yeast strain more recently derived from the wild would undergo chronological aging in a manner similar to a laboratory yeast strain. The results presented here suggest that this is the case. In all of the aging conditions examined, the vineyard strain RM11 and its haploid derivatives showed similar chronological aging properties as the laboratory strain BY4743 and its haploid derivatives. This includes significant extension of chronological life span from dietary restriction, buffering of the culture medium, or growth in rich YEPD medium.

Prior studies in nematodes and mice have shown that wild-derived strains can have life spans that differ significantly from laboratory strains, and at least one wild-derived mouse strain fails to have its median life span extended by dietary restriction [18], [19]. Such differences may result from evolution in the laboratory strains, which are subjected to selective pressures different from those in the wild. In particular, selection for maximal growth and reproduction under nutrient rich conditions is a common hallmark of laboratory life. It is interesting therefore that RM11 and BY4743 had generally similar chronological aging properties across a variety of different conditions. One possibility is that RM11 has already undergone substantial laboratory selection since its isolation from the vineyard. Arguing against this are the substantial differences between RM11 and BY4743 at the nucleotide, mRNA, and proteomic levels [20], [21], [22], [23]. Also arguing against this is the report that RM11 has a replicative life span that is significantly greater than BY4743 and several other laboratory yeast strains [24].

We have previously reported that acidification of the culture medium and accumulation of acetic acid can limit chronological life span for laboratory strains aged in synthetic defined medium [12]. Dietary restriction or buffering of the culture medium to pH 6.0 extended chronological life span in the diploid BY4743, as well as the haploid W303AR5, PSY316AT, and DBY746 strains in that study [12]. Here we have shown that similar effects are observed in RM11 diploid and haploid cells. This finding is consistent with a prior report in which it was shown that maintaining the pH of the culture medium between 6.5–7 extended the chronological life span of cells isolated from grapes that were morphologically similar to budding yeast [13].

This study also further strengthens the link between culture acidification and chronological life span by finding a significant inverse correlation between culture pH after either 2 or 4 days of growth and chronological longevity for both BY4743 and RM11 cells aged in media containing different carbon sources. These data are consistent with our recent report that deletion mutants isolated from a screen for reduced culture acidification are more likely to have extended chronological life span, relative to randomly selected deletion mutants [25]. Our data also support a prior study which reported that cells aged with galactose or fructose as the carbon source have their life span extended by dietary restriction, while cells aged in non-fermentable carbon sources have extended chronological life span even under non-dietary restriction conditions, relative to cells grown in glucose cultures [26].

The data presented here strongly suggest that a similar mechanism of aging limits the chronological life span of both diploid and haploid versions of laboratory and vineyard yeast strains. Although we have not examined additional features of chronological aging, such as elevated levels of reactive oxygen species and induction of the yeast apoptotic-like response, we predict that these will also be similar in the vineyard and laboratory yeast strains. Despite the fact that many questions remain to be resolved regarding the optimal conditions for performing chronological life span experiments in yeast and the ultimate utility of this paradigm as a model for aging in multicellular eukaryotes [12], [14], [27], [28], this study suggests that the processes governing chronological life span are shared between laboratory and wild yeast and that conclusions drawn from yeast chronological aging studies, at least under the conditions tested, are unlikely to be the result of artificial selection for growth under laboratory conditions.

## Materials and Methods

### Yeast strains and media

The strains used in this study are described in **Table 5**. Chronological life span assays were performed as previously described using the Bioscreen C MBR automated shaker/incubator/plate reader [3], [4], [29]. All aging cultures were initiated by seeding a 5 ml liquid culture of YEPD with a single colony from a freshly streaked strain grown on YEPD agar at 30°C. A 1∶100 dilution of the YEPD culture was made into synthetic complete (SC) medium, containing 2% glucose, unless otherwise noted. Basic medium is 1.7 g/L Yeast Nitrogen Base (−AA/−AS) (BD Difco™) and 5 g/L (NH_4_)_2_SO_4_. Components of the synthetic complete medium used in this study have been described elsewhere in detail [4] and are shown in **Table 6**. All strain auxotrophies were compensated with a four-fold excess of amino acids. Cultures were grown and aged in a roller drum enclosed in a water-jacketed incubator at 30°C. YEPD was 20 g/L Bacto Peptone and 10 g/L Yeast Extract (BD Difco™), supplemented with glucose at the indicated concentrations. For alternative carbon source experiments, the glucose was replaced with the indicated carbon source at the indicated concentration. For growth in buffered medium, a citrate phosphate buffer (64.2 mM Na_2_HPO_4_ and 17.9 mM citric acid, pH 6.0) adjusted to pH 6.0 was added to the medium prior to inoculation.

**Table 5:** Strains used in this study.

Strain	Genotype
BY4741	*MAT* **a** *his3*Δ*1 leu2*Δ*0 ura3*Δ*0 met15*Δ*0*
BY4742	*MAT*α *his3*Δ*1 leu2*Δ*0 ura3*Δ*0 lys2*Δ*0*
BY4743	*MAT* **a**/α *his3*Δ*1 leu2*Δ*0 ura3*Δ*0*
RM11-1a	*MAT* **a** *leu2*Δ*0 ura3*Δ*0 ho::KAN*
RM11α	*MAT*α *lys2*Δ*0 ura3*Δ*0 ho::KAN*
RM11 Diploid	*MAT* **a**/α *ura3*Δ*0 ho::KAN*

**Table 6:** Media composition for chronological aging experiments.

Component	Concentration (g/L)
D-glucose	20
Yeast Nitrogen Base (−AA/AS)	1.7
(NH_4_)_2_SO_4_	5.0
Adenine	0.04
L-Arginine	0.02
L-Aspartic Acid	0.1
L-Glutamic Acid	0.1
L-Histidine	0.1
L-Leucine	0.3
L-Lysine	0.03
L-Methionine	0.02
L-Phenylalanine	0.05
L-Serine	0.375
L-Threonine	0.2
L-Tryptophan	0.04
L-Tyrosine	0.03
L-Valine	0.15
Uracil	0.1

Standard synthetic complete medium is composed of the following:

This medium contains excess concentrations of leucine, histidine, and uracil to compensate for auxotrophies present in the laboratory strains used in this study. For experiments using BY4741, the concentration of methionine was increased to 0.1 g/L; for experiments using BY4742, the concentration of lysine was increased to 0.15 g/L. The concentration of glucose or other carbon sources described in the text were varied as indicated. A citrate phosphate buffer (64.2 mM Na2HPO4 and 17.9 mM citric acid) adjusted to pH 6.0 was added to the standard synthetic complete recipe for experiments using buffered SC medium.

YEPD medium contained 2% bacto peptone and 1% yeast extract supplemented with filter-sterilized glucose at 2%.

### Growth rate analysis

Doubling times were determined using a Bioscreen C MBR machine (Growth Curves USA) as previously described using the Yeast Outgrowth Data Analyzer (YODA) [30]. Reported doubling times in 30°C YEPD are taken from interval readings from the OD_420–580_ = 0.2–0.6 range of maximum growth rate. Significance was tested with a two-tailed Student's t-test of mutant doubling times compared to wild type doubling times. Each experiment was performed in biological triplicates per run (three cultures, three Bioscreen C Honeycomb Plate wells) and the runs were performed on at least three biological replicate cultures (independently grown from different colonies on different days).

### pH Determinations

Aging cultures were prepared as described above, with a 1∶100 dilution of a YEPD culture being inoculated into synthetic medium containing the appropriate carbon source. Cultures were left for either two or four days, and pH was determined using an Accumet XL 15 pH meter (Fisher Scientific). Between readings, the meter was rinsed with ethanol, sterile deionized water, and patted dry with a laboratory tissue wipe.

### Chronological lifespan analysis

Unless otherwise stated, chronological life span was determined using a Bioscreen C MBR automated incubator/plate reader to monitor the outgrowth kinetics of chronologically aged cultures in a synthetic complete medium supplemented with 2% glucose, as previously described [3], [4], [29]. Chronological viability was calculated from growth curves of aging cultures using the Yeast Outgrowth Data Analyzer (YODA, www.sageweb.org/yoda) [30]. The survival integrals were calculated using the YODA “cleaned” algorithm on YODA and the doubling time was calculated by the “interval” method. The calculation parameters were as follows: Threshold ODs (Min = 0.100, Max = 2.000); Doubling Time Interval OD (Min = 0.200, Max = 0.500); Doubling Time Adjustment (Delay OD = 0.500, Slope = 0.0261, Min Delay (sec) = 500); Survival Time Shift (OD = 0.300). Survival integral (SI) is defined as the area under the mortality curve and provides a quantitative measure of the chronological life span that allows for statistical analysis between experimental and control groups [3], [29]. A Student's T-test was used to calculate p-values. For all experiments, outgrowth data was normalized to the initial time point collected on the second day of chronological aging. In all experiments, data for at least 3 biological replicates are shown, except for RM 0.05% glucose in both **a** and *α* mating types in which only two biological replicates were analyzed due to contamination of the third replicate.
